# A 65‐year‐old woman with ALS and bilateral precentral motor band sign

**DOI:** 10.1002/ccr3.9014

**Published:** 2024-05-29

**Authors:** Sadegh Ghaderi, Seyed Amir Hossein Batouli, Sanjay Kalra, Sana Mohammadi, Farzad Fatehi

**Affiliations:** ^1^ Department of Neuroscience and Addiction Studies, School of Advanced Technologies in Medicine Tehran University of Medical Sciences Tehran Iran; ^2^ Neuromuscular Research Center, Department of Neurology, Shariati Hospital Tehran University of Medical Sciences Tehran Iran; ^3^ Neuroscience and Mental Health Institute University of Alberta Edmonton Alberta Canada; ^4^ Division of Neurology, Department of Medicine University of Alberta Edmonton Alberta Canada; ^5^ Neurology Department University Hospitals of Leicester NHS Trust Leicester UK

**Keywords:** amyotrophic lateral sclerosis, biomarker, iron, motor band sign, quantitative susceptibility mapping, susceptibility‐weighted imaging

## Abstract

Advanced MRI techniques, including SWI, MinIP, and QSM, are instrumental in detecting the “motor band sign” in ALS, aiding in the early diagnosis and assessment of upper motor neuron involvement, which is critical for therapeutic interventions.

## Graphical abstract

MRI with advanced techniques such as QSM reveals a distinct “motor band sign” in a patient with ALS, indicating upper motor neuron involvement.

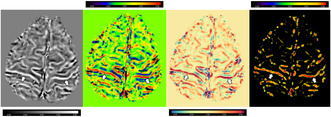

## 1 CLINICAL STUDY

### 1.1 Case presentation and imaging

A 65‐year‐old woman who was illiterate and worked as a housewife and gardener was referred to the amyotrophic lateral sclerosis (ALS) clinic. She was diagnosed with clinically definite limb‐onset ALS using the Awaji criteria. The initial symptoms appeared 31 months prior to imaging. At the time of imaging, her ALSFRS‐R and BMI scores were 43 and 42.44 kg/m^2^, respectively. Imaging, including susceptibility‐weighted imaging (SWI) and minimum intensity projection (MinIP) protocols and acquisitions, as well as post‐processing for quantitative susceptibility mapping (QSM) analyses, was conducted based on our previous study.^1^ SWI visually displays tissue magnetic field variations using magnitude and phase information, whereas QSM quantifies the underlying magnetic susceptibilities that cause these variations.^1^ However, in this study, we used a visual map of the susceptibility to precentral MBS.

## 2 RESULTS AND DISCUSSION

**FIGURE 1:**
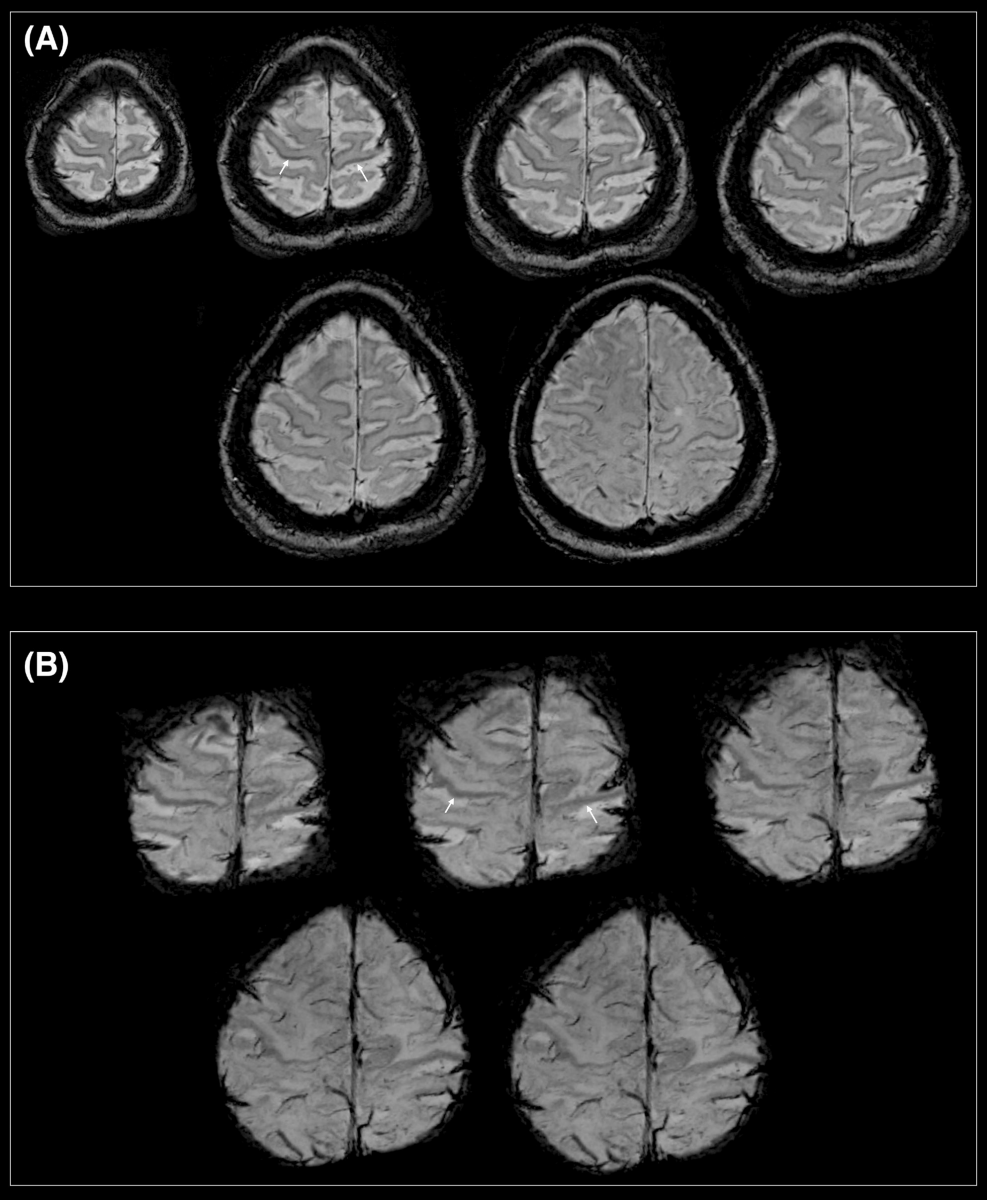
Motor band signs (white arrows) showing bilateral hypointense signals in the precentral region on (A) susceptibility‐weighted imaging (SWI) and (B) minimum intensity projection (MinIP) images in a patient diagnosed with ALS.

**FIGURE 2:**
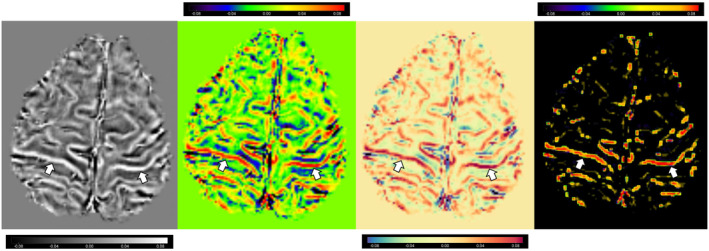
Quantitative susceptibility mapping images (using four different filters with MRIcroGL) of the bilateral precentral gyrus showing motor‐band signs (white arrows).

MRI revealed a bilateral decreased signal (hypointensity) on SWI, MinIP, and QSM images, consistent along the precentral gyri, which is referred to as the “motor band sign” or “black ribbon sign”^1^, ^2^ (Figures 1 and 2). The precentral motor band sign is a suggestive and sensitive biomarker for motor neuron diseases (MNDs) such as ALS^1^ and other neurological disorders such as Huntington's disease.^3^ This sign is believed to result from the accumulation of iron in the motor cortex owing to oxidative stress and microglia‐driven neuroinflammation.^1^ MRI with MinIP and/or SWI and post‐processing techniques such as QSM may play a vital role in the diagnosis of rare cases of ALS and in assessing UMN involvement in therapeutic trials.^1^, ^2^ Thus, advanced MRIs, such as T2*‐weighted, transverse relaxation rates, SWI, and QSM, have been proposed to detect radiological markers of upper motor neuron degeneration (UMN) in ALS.^1^, ^2^

Close monitoring for emerging MND should be considered when MBS is present on the SWI/QSM. In research and clinical settings, SWI and QSM are useful for detecting cortical neuroimaging biomarkers of UMN degeneration in MNDs.^1^, ^2^ By combining structural, functional, metabolic, and quantitative techniques, multimodal MRI can provide the best imaging biomarkers. However, feasible protocols for clinical use must be optimized.

## AUTHOR CONTRIBUTIONS

**Sadegh Ghaderi:** Conceptualization; data curation; formal analysis; investigation; methodology; project administration; resources; software; validation; visualization; writing – original draft; writing – review and editing. **Seyed Amir Hossein Batouli:** Conceptualization; investigation; project administration; validation; visualization; writing – review and editing. **Sanjay Kalra:** Conceptualization; investigation; project administration; validation; visualization; writing – review and editing. **Sana Mohammadi:** Data curation; formal analysis; investigation; methodology; resources; software; validation; writing – original draft; writing – review and editing. **Farzad Fatehi:** Conceptualization; investigation; methodology; project administration; supervision; validation; visualization; writing – original draft; writing – review and editing.

## 3 FUNDING INFORMATION

This research received no specific grant from any funding agency in the public, commercial, or not‐for‐profit sectors.

## CONFLICT OF INTEREST STATEMENT

The authors declare that they have no conflict of interest.

## ETHICAL STATEMENT

The study was approved by the Ethics Committee of the Tehran University of Medical Sciences (Ethical Code: IR.TUMS.MEDICINE.REC.1400.1173). We obtained written consent from our patient, who may have an identifiable image or data.

## CONSENT

Written informed consent was obtained from the patient to publish this report in accordance with the journal's patient consent policy.

## Data Availability

The data that support the findings of this study are available from the corresponding author upon reasonable request.
